# Post-epidemic awareness and knowledge of Lassa fever among residents in affected community in Ibadan, Oyo State, Nigeria

**DOI:** 10.14202/vetworld.2018.1059-1063

**Published:** 2018-08-04

**Authors:** E. J. Awosanya

**Affiliations:** Department of Veterinary Public Health and Preventive Medicine, Faculty of Veterinary Medicine, University of Ibadan, Ibadan, Oyo State, Nigeria

**Keywords:** IEC materials, knowledge, Lassa fever, outbreaks

## Abstract

**Aim:**

An outbreak of Lassa fever occurred in Ibadan with a case fatality rate of 50% in 2012. Awareness creation and sensitization is a known disease prevention and control strategy. An assessment of the awareness level and knowledge of Lassa fever in the affected community and a nearby university community was done to aid the development of effective information, education, and communication (IEC) material adaptable to the affected community.

**Materials and Methods:**

A semi-structured questionnaire was used to obtain the data about awareness and knowledge of Lassa fever from 130 respondents. Descriptive statistics and statistical differences between categorical variables were done using Fisher’s exact test at 5% significant level.

**Results:**

Respondents’ age was 29.9 ± 10.9 years. Awareness level in the affected and university communities was 42 (65%) and 55 (85%), respectively (p=0.02). The most reported source of awareness was the television and radio (59.8%). Only 33.1% of all respondents had good knowledge of the clinical symptoms. Most (68.5%) of the respondents knew rat as the reservoir: However, 56.9% and 80.0% of respondents from the affected and university communities, respectively, had this knowledge (p=0.01). About one-third (30.0%) of the respondents had good knowledge of preventive measures: 18.5% and 41.5% from affected and university communities, respectively (p=0.01).

**Conclusion:**

Knowledge of respondents on Lassa fever symptoms, reservoir, and preventive measures was low in the affected community; the IEC material was developed to address the knowledge gaps. Awareness was also intensified in the affected community.

## Introduction

Lassa fever is one of the viral hemorrhagic fevers caused by a single-stranded RNA virus beloning to the virus family Arenaviridae. Lassa fever presents with gradual onset of fever, malaise, headache, sore throat, muscle pain, chest pain, nausea, vomiting, diarrhea, cough, and abdominal pain, which may progress to facial edema, mucosal bleeding, disorientation, coma, and death in the late stages. The incubation period of Lassa fever ranges from 6 to 21 days [1]. The case fatality rate (CFR) may range from 1% to 15% [1] but could exceed 50% depending on the patient status [2-4]. There could be a direct transmission of the disease to humans through ingestion of food and food materials contaminated by the feces and/or urine of the mammalian reservoir of the Lassa virus, a peridomestic multimammate rat, *Mastomys natalensis*. The disease could also spread from person to person, especially, in nosocomial cases through contact with secretions and excretions of infected persons [1]. About 80% of human infections are asymptomatic [1,3].

The first outbreak of Lassa fever in Nigeria was in 1969 in a village called Lassa in Borno State [5], and the disease has assumed an endemic status [1]. About two-third of the 36 states in Nigeria are endemic. There appears to be a seasonal pattern in the outbreak of Lassa fever in Nigeria, with most cases occurring in the dry season. Within the past 7 years, Nigeria reported between 18 and 201 confirmed cases of Lassa fever annually, with annual CFR of between 24% and 79% among confirmed cases [6].

A single case of Lassa fever is regarded as an outbreak, and a suspected case of Lassa fever is defined as illness with gradual onset with one or more of the following: Malaise, fever, headache, sore throat, cough, nausea, vomiting, diarrhea, myalgia, chest pain hearing loss, and a history of contact with excreta of rodents or with a case of Lassa fever, while a confirmed case of Lassa fever is a suspected case that is laboratory confirmed (positive IgM antibody, PCR, or virus isolation) or epidemiologically linked to a laboratory-confirmed case [7].

In August 2012, Oyo State had four suspected cases of Lassa fever; two were laboratory-confirmed cases and one fatality [6]. As part of the interventions, an assessment of the awareness level and knowledge of Lassa fever of the affected local community and a nearby unaffected university community was done to develop an effective information education and communication material that can be adapted to the affected local community.

This study, therefore, aimed to determine the awareness level immediately after the outbreak of Lassa fever and to identify knowledge gaps in the affected local community.

## Methods

### Ethical approval and informed consent

The study was part of outbreak response, so the ethical approval was not necessary. However, respondents who do not wish to participate were respected and not denied of the common right on information dissemination. Confidentiality of the collected data was maintained. Verbal informed consent was obtained from each participant.

### Study site and setting

The study site was Ibadan in Oyo State. Ibadan is located on latitude 7.3877800 and longitude 3.8963900 in decimal coordinates. The human population of Ibadan is about 2,559,853 of the 5,580,894 in Oyo State [8]. Ibadan has two tertiary hospitals, 18 secondary and 244 primary health facilities [9]. Ibadan is an agrarian community with some commercial and industrial activities. The inhabitants are diverse with the Yoruba Ethnic Group in the majority. The Yorubas have a predilection for living in high-density urban centers [10].

### Study period

The study was conducted from September to December 2012.

### Study design

This study was a cross-sectional survey.

### Study population

The respondents were residents from the affected local community and a university community with no history of Lassa fever outbreak. Half of the total respondents were each from affected local community and a university community without history of Lassa fever outbreak.

### Sample size and sampling

A total of 130 respondents were engaged. The formula for a cross-sectional survey was used [11] with the assumption that 50% of the community members were aware of Lassa fever and had good knowledge of it and a margin of error of 10%. To minimize sampling bias, the respondents were selected using transect-trained enumerators (2) moved in all directions, and in any street, every other household was selected until the sample size was completed. More so, it was ensured that the calculated true population mean age of 24.5 years for Ibadan [8] falls within one standard deviation from the sampled respondents’ mean age.

### Data collection and reliability

A semi-structured questionnaire was used to obtain the data on demography, awareness, and knowledge of Lassa fever. The questionnaire was pretested and interviewer administered to the respondents. The knowledge scale on Lassa fever has good internal consistency, with a Cronbach’s alpha coefficient of 0.75.

### Data analysis

The data were entered into Excel spreadsheet 2010 and analyzed using Epi-Info version 3.5.4. Knowledge of clinical symptoms, general preventive measures, and personal protective measures was on a scale of four; a score of two and above is graded as good. In addition, ability to know that spread of the Lassa fever is through contact with the secretion or excretion from an infected person and that rat can transmit the Lassa virus is graded as good. Descriptive statistics were done, and significant differences between categorical variables were assessed using Fisher’s exact test. The level of significance was 5% at 95% confidence interval.

## Results

**Figure-1:**
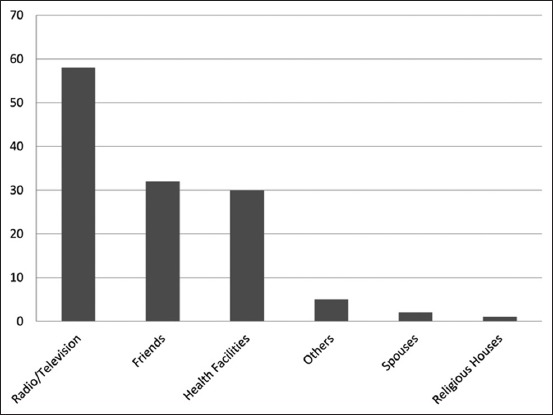
Source of awareness on Lassa fever among respondents in both affected local and unaffected university communities, Ibadan, Nigeria 2012.

The mean age of the respondents was 29.9±10.9 years, 77 (59.2%) were female. Of the 130 respondents, most (66.9%) had tertiary education (Table-1). The overall awareness level of Lassa fever was 74.6%. About 65% (42 of 65) of respondents from the affected local community and about 85% (55 of 65) of respondents from the unaffected university community were aware of Lassa fever. The difference in awareness level was statistically significant at p=0.02 (Table-2). Only 33.1% of the total respondents had good knowledge of the clinical symptoms. The knowledge of clinical symptoms from the affected and university communities was 29.2% and 36.9%, respectively. The difference was, however, not statistically significant (p>0.05). Most (68.5%) of all respondents had good knowledge of the reservoir as rat. The knowledge of the reservoir from the affected and university communities was 56.9% and 80.0%, respectively. The difference was statistically significant (p=0.01). About one-third (30.0%) of the total respondents had a good knowledge of preventive measures. The knowledge of preventive measures from the affected and university communities was 18.5% and 41.5%, respectively. The difference was statistically significant (p=0.01) (Table-2). Of the 97 respondents who were aware of Lassa fever, the most reported source of awareness was the television and radio (59.8%), while the least reported source was religious houses (1.0%) (Figure-1). Residents from both the affected (61.9% [26 of 42]) and university (58.2% [32 of 55]) communities reported the television and radio as the most source of information; however, the difference was not statistically significant between the two communities. Friends (47.3% [26 of 55]) were the second most source of information among residents from the university community and the third most source of information (14.3% [6 of 42]) in the affected local community; the difference was, however, statistically significant (p=0.02) between the communities. On what to do should they have persistent fever, sore throat, persistent headache for more than three days, most respondents both from the affected (72.3% [47 of 65]) and university (80.0% [52 of 65]) communities would visit a health center. However, though not statistically significant, it is noteworthy that respondents from the affected local community are more likely to visit other places, such as patent or drug store, herbalist, or use of herbs, while less likely to self-medicate or visit religious houses than respondents from the university community (Table-3).

**Table-1:** Demographic characteristics of respondents in the affected local and unaffected university communities, Ibadan, Nigeria 2012.

Variables	Affected local community n=65 (%)	Unaffected University community n=65 (%)
Age (years)		
<18	0 (0)	1 (1.5)
18-22	4 (6.2)	35 (53.9)
23-27	9 (13.8)	21 (32.3)
28-32	16 (24.6)	8 (12.3)
33-37	8 (12.3)	0 (0)
>38	28 (43.1)	0 (0)
Gender		
Female	37 (56.9)	40 (61.5)
Male	28 (43.1)	25 (38.5)
Educational level		
None	1 (1.5)	0 (0)
Primary	7 (10.8)	0 (0)
Secondary	35 (53.9)	0 (0)
Tertiary	22 (33.8)	65 (100)

**Table-2:** Test of significance in awareness level and knowledge of Lassa fever between affected local and unaffected university communities, Ibadan, Nigeria 2012.

Variables	Affected local community n=65 (%)	Unaffected university community n=65 (%)	Odds ratio (95% CI)	p-value
Awareness level				
Yes	42 (64.6)	55 (84.6)	0.3 (0.1-0.8)	0.02*
No	23 (35.4)	10 (15.4)		
Knowledge of symptoms				
Good	19 (29.2)	24 (36.9)	0.7 (0.3-1.5)	0.46
Fair	46 (70.8)	41 (63.1)		
Knowledge of spread				
Good	9 (13.8)	3 (4.6)	3.3 (0.9-12.9)	0.13
Fair	56 (86.2)	62 (95.4)		
Knowledge of prevention				
Good	12 (18.5)	27 (41.5)	0.3 (0.1-0.7)	0.01*
Fair	53 (81.5)	38 (58.5)		
Knowledge of role of rat in transmission				
Good	37 (56.9)	52 (80.0)	0.3 (0.2-0.7)	0.01*
Fair	28 (43.1)	13 (20.0)		
Knowledge of what to do personally to avoid contracting Lassa fever				
Good	15 (23.1)	10 (15.4)	1.7 (0.7-4.0)	0.37
Fair	50 (76.9)	55 (84.6)		

* Significant atP*<*0.05. CI=Confidence interval

**Table-3:** Bivariate analysis of source of awareness and what action will be taken in case of index of suspicion for Lassa fever among respondents in affected local and unaffected university communities, Ibadan, Nigeria 2012.

Variables with multiple responses	Affected local community n (%)	Unaffected university community n (%)	Odds ratio (95% CI)	p-value
Source of awareness				
Radio/TV	26 (55.3)	32 (39.5)	Ref.	
Health workers	11 (23.4)	19 (23.5)	0.7 (0.31.9)	0.61
Friends	6 (12.8)	26 (32.1)	0.3 (0.10.9)	0.02*
Religious houses, spouse, and others	4 (8.5)	4 (4.9)	1.2 (0.27.3)	1.00
Action to be taken on index of suspicion for Lassa fever				
Visit to nearby health center	47 (53.4)	52 (61.2)	Ref.	
Visit to a patent/drug store	16 (18.2)	7 (8.2)	2.5 (0.97.9)	0.09
Visit to a herbalist/use herbs	7 (7.9)	2 (2.4)	3.9 (0.739.6)	0.16
Visit to a religious house	3 (3.4)	7 (8.2)	0.5 (0.12.2)	0.47
Selfmedication	13 (14.8)	16 (18.8)	0.9 (0.42.2)	0.97
Others	2 (2.3)	1 (1.2)	2.2 (0.1133)	0.94

* Significant atP*<*0.05. CI=Confidence interval

## Discussion

The overall awareness level of Lassa fever by respondents was moderately high, with respondents from the university community 3 times more likely to be aware than local community. Level of education has been associated with awareness level and knowledge of Lassa fever [12]. Low level of awareness on Lassa fever is often reported from members of local communities [12-15]. However, Reuben and Gyar [16] reported a higher level of awareness in a local community probably due to intensified sensitization and the extent of endemicity of Lassa fever in that locality. The most reported source of awareness was the radio and television similar to the reports of Ilesanmi *et al*. [13], Olalekan [14], and Asogun *et al*. [12] in local communities. However, among respondents from the university community, friends were equally found to play a significant role as a source of awareness on Lassa fever. Friends have been reported to play a vital role in information dissemination among students of secondary and tertiary learning [17,18]. However, it has been reported that information dissemination on a thematic issue is more effective if several media are adopted than just a particular channel [19].

More than half of the respondents had knowledge of the role of rat (*Mastomys* spp.) in the transmission of the Lassa virus, similar to the findings of Akinbodewa *et al*. [20] and Asogun *et al*. [12]. However, respondents from the university community were 3 times more likely to have good knowledge of the role of rat in Lassa virus transmission than respondents from the affected local community. The knowledge of the respondents on general prevention of Lassa fever in both local and university communities was low, similar to the report of Olalekan [14]. In a similar pattern, respondents from the university community were 3 times more likely to have good knowledge of general prevention of Lassa fever than those from local communities. Although the questionnaire was interviewer administered to respondents in their local language, the higher level of education among respondents from the university community could have accounted for the differences in knowledge levels [12]. Another possible contributory factor to the differences in knowledge levels could be the reduced frequency with which information on Lassa fever is heard on either the radio or television due to an erratic power supply which is more of a challenge in local communities than the university community. In general, low knowledge of the early symptoms of Lassa fever was observed among the respondents similar to the report of Akinbodewa *et al*. [20]. Although the early symptoms of Lassa fever are similar to that of malaria and other viral hemorrhagic fevers, the knowledge of Lassa clinical presentations could assist an affected individual in seeking health-care intervention early. Early treatment with ribavirin is effective as a post-exposure prophylactic though with possible side effects [21].

It is worth mentioning that respondents from affected local community were 3 times more likely to have good knowledge of the manner of spread of Lassa fever and 2 times more likely to have good knowledge of how to personally protect oneself from contracting Lassa infection than respondents from the unaffected university community, though not significantly so, maybe due to recent experience of Lassa fever outbreak. Experience sometimes may positively influence knowledge acquisition [22]. In addition, the more likelihood of respondents in affected local community than the university community to visit other places such as drug or patent stores and herbalist centers aside health-care centers for medical attention should inform health workers or researchers of other places for active case search, surveillance, and route of information dissemination when working among such study population. Uzochukwu and Onwujekwe [23] observed a similar trend in the health-seeking behavior of respondents in the treatment and diagnosis of malaria, a febrile illness which presents with similar symptoms with the early stage of Lassa fever. The observation also lays credence to approach information dissemination on a thematic issue through the use of the combination of channels for IEC to be effective [19].

## Conclusion

The knowledge gaps identified were the low knowledge level on the role of the rat in the transmission of Lassa virus, general preventive measures, and early symptoms of Lassa fever among affected local respondents which were taken into consideration in the development of the IEC materials in the affected local community. The importance of seeking immediate health care in health-care facilities should an individual have persistent fever, sore throat, and persistent headache for more than 3 days was also stressed in the jingle developed The message content of IEC materials should be developed not only to acquaint the people but also to provide the needed knowledge for action.

## Authors’ Contributions

EJA conceived the study, analyzed the data, drafted, and approved the final manuscript.
